# Dysregulation of Glutathione Homeostasis in Neurodegenerative Diseases

**DOI:** 10.3390/nu4101399

**Published:** 2012-10-09

**Authors:** William M. Johnson, Amy L. Wilson-Delfosse, John. J. Mieyal

**Affiliations:** 1 Department of Pharmacology, Case Western Reserve University, 2109 Adelbert Rd., Cleveland, OH 44106, USA; Email: wmj18@case.edu (W.M.J.); axw41@case.edu (A.L.W.-D.); 2 Louis B. Stokes Veterans Affairs Medical Research Center, 10701 East Blvd, Cleveland, OH 44106, USA

**Keywords:** glutathione, *N*-acetylcysteine, oxidative stress, redox signaling, neurodegenerative diseases, apoptosis, protein aggregation, glutathionylation, nitrosylation

## Abstract

Dysregulation of glutathione homeostasis and alterations in glutathione-dependent enzyme activities are increasingly implicated in the induction and progression of neurodegenerative diseases, including Alzheimer’s, Parkinson’s and Huntington’s diseases, amyotrophic lateral sclerosis, and Friedreich’s ataxia. In this review background is provided on the steady-state synthesis, regulation, and transport of glutathione, with primary focus on the brain. A brief overview is presented on the distinct but vital roles of glutathione in cellular maintenance and survival, and on the functions of key glutathione-dependent enzymes. Major contributors to initiation and progression of neurodegenerative diseases are considered, including oxidative stress, protein misfolding, and protein aggregation. In each case examples of key regulatory mechanisms are identified that are sensitive to changes in glutathione redox status and/or in the activities of glutathione-dependent enzymes. Mechanisms of dysregulation of glutathione and/or glutathione-dependent enzymes are discussed that are implicated in pathogenesis of each neurodegenerative disease. Limitations in information or interpretation are identified, and possible avenues for further research are described with an aim to elucidating novel targets for therapeutic interventions. The pros and cons of administration of *N*-acetylcysteine or glutathione as therapeutic agents for neurodegenerative diseases, as well as the potential utility of serum glutathione as a biomarker, are critically evaluated.

## Abbreviations

AD: Alzheimer’s disease
ALS: amyotrophic lateral sclerosis, also known as Lou Gehrig’s disease
ARE: Antioxidant Response Element
EAAC1: Excitatory amino acid transporter C1
EAAT: Excitatory amino acid transporter
ERE: Electrophile Response Element
FA: Friedreich’s axtaia
GCLC (heavy subunit of GCS), GCS: γ-glutamylcysteine synthetase
Grx: glutaredoxin
GSSG: glutathione disulfide
GPx: glutathione peroxidase
GST: glutathione *S*-transferase
GS: glutathione synthetase
GR: glutathione reductase
HD: Huntington’s disease
MS: multiple sclerosis
MPTP: 1-methyl-4-phenyl-1,2,3,6-tetrahydropyridine
PD: Parkinson’s disease
RNS: reactive nitrogen species
ROS: reactive oxygen species
GSH: reduced glutathione
Xc^−^: cystine/glutamate transport system

## 1. Introduction

Glutathione (GSH) is a vital constituent of cells throughout the body, acting as a redox buffer, and as cofactor for signal transduction, antioxidant defense, and electrophile defense, especially in the brain. Thus, dysregulation of GSH homeostasis and deactivation of GSH-dependent enzymes are believed to contribute to initiation and progression of neurodegenerative diseases, the focus of this review. Parkinson’s disease, Alzheimer’s disease, Huntington’s disease, Amyotrophic lateral sclerosis, and Friedreich’s ataxia are featured. Multiple sclerosis ultimately leads to neurodegeneration, however it is primarily considered to be an autoimmune disease, so it is not discussed extensively herein. The review presents a critical overview of the ways that alterations in GSH-dependent biochemistry and cell biology may contribute to neurodegenerative diseases, considering limitations in available information and suggesting future studies. To provide perspective, the steady-state synthesis, regulation, and transport of GSH are described with emphasis on these processes in the brain. Then the functions of GSH as redox buffer, nucleophilic scavenger of electrophilic compounds, and signal transduction agent are presented. Building on this foundation, the potential role of impairment of GSH-dependent functions is evaluated in the context of major contributing mechanisms of initiation and progression of the neurodegenerative diseases, including oxidative stress, deactivation of key enzymes, disruption of thiol homeostasis and signaling pathways, protein misfolding, and protein aggregation. Finally, the potential utility of GSH as a therapeutic agent and/or biomarker is evaluated. 

## 2. Intracellular Synthesis and Transport of GSH in Brain

GSH is synthesized in cells, with intracellular concentrations ranging from 0.2 to 10 mM [1]. The typical concentration of GSH in most cells, including neurons is around 1–2 mM. It is important to note that the cerebral spinal fluid concentration of GSH is approximately 4 μM. This corresponds approximately to a 250–500× lower concentration than intracellular GSH levels. Thus intracellular synthesis is necessary for maintaining the large concentration difference. The rate-limiting step of GSH synthesis is the formation of the amide linkage between the gamma-carboxyl moiety of glutamic acid and the amino moiety of cysteine. The rate at which GSH is synthesized is based on both the activity of the enzyme γ-glutamylcysteine synthetase (GCS) and the availability of cysteine. Completing the synthesis of GSH, glutathione synthetase (GS) catalyzes the conversion of the γ-GluCys dipeptide to GSH by addition of glycine. The enzymes involved in GSH synthesis are controlled by multiple mechanisms both pre/post transcriptionally. For example, the human *GCLC* (subunit of GCS) promoter contains multiple consensus sequences for transcription factors, including NFκB, AP-1 and antioxidant response/electrophile response elements (ARE/ERE) [2,3,4]. Additionally, Liu *et al*. found in L2 lung cells, 4-hdroxy-2-nonenal increased mRNA stability for both subunits of GCS that resulted in increased activity of GCS [5]. Furthermore, regulation of GCS activity can be modulated via phosphorylation. Protein kinase A, Protein kinase C, and Ca^2+^/calmodulin-dependent kinase II have all been reported to phosphorylate (resulting in inhibition) GCS isolated from rat kidneys [6]. Thus, GSH synthesis can be controlled through a variety of mechanisms, accentuating the importance of GSH within the cell. The descriptions above provide a concise insight into the complex regulation of GSH synthesis. For more detailed reviews of regulation of GSH synthesis see Lu [7]; Aoyama *et al.* [8]. 

As mentioned above, the synthesis of GSH is also dependent upon the availability of the rate limiting amino acid cysteine. In neuronal tissue, cells must take up a majority of the cysteine they use in GSH production from the extracellular space. Cysteine incorporation into neurons and astrocytes occurs via two primary mechanisms. First, neurons primarily use excitatory amino acid transporters (EAATs) to bring cysteine into cells [9]. Secondly, astrocytes use the cystine/glutamate transporter (Xc^−^) to increase intracellular levels of cysteine through the uptake of cysteine, the oxidized form of cysteine. In addition to importation of extracellular cyst(e)ine, cells can generate cysteine using intracellular polypeptides as precursors in a process called transsulfuration. However, the relative importance of uptake mechanisms and transsulfuration is cell-type dependent, as described briefly below for the specific brain cell types, along with references to more in-depth discussions. 

**Figure 1:**
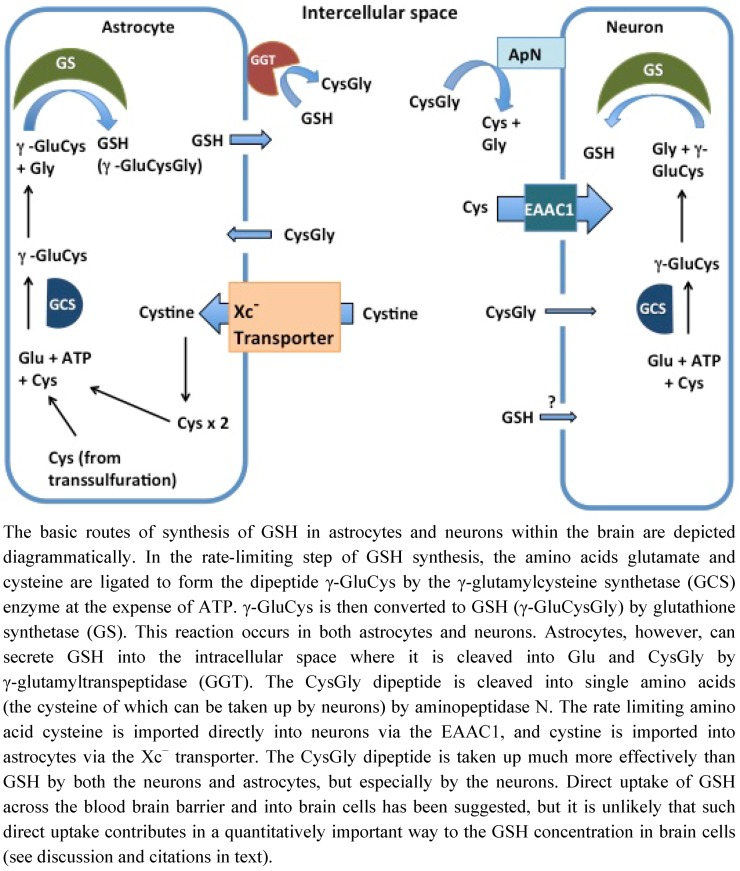
Production of glutathione (GSH) in astrocytes and neurons.

Astrocytes are reported to have higher GSH levels than neurons and also to have the ability to secrete GSH into the extracellular space [10]. This secretion of GSH serves as a precursor supplier for other brain cells. In the intracellular space, the secreted GSH is cleaved into Glu and CysGly by γ-glutamyltranspeptidase (GGT), and the CysGly dipeptide is then cleaved into Cys and Gly by the ectopeptidase aminopeptidase N [11] (Figure 1). The free Cys can then be taken up and used for GSH synthesis by neurons or oxidized in the extracellular space into cystine to be re-used by the astrocytes. Additionally, GGT can transfer the γ-glutamyl moiety to an amino acid acceptor; one of these acceptors is cystine [12]. The resulting γ-glutamylcystine has been shown to increase GSH content *in vivo*, as mice injected with radiolabeled γ-glutamylcystine showed almost a doubling of kidney GSH levels [13]. A more recent study using primary astrocytes also investigated the mechanism of γ-glutamylcystine uptake. However, this study found that pretreatment of the astrocytes with acivicin (which inactivates GGT) resulted in no change in intracellular GSH levels compared to control cells treated with cystine in the absence of acivicin [14]. This led the authors to state, “Since the cystine effect was not affected by inhibiting γ-glutamyl transpeptidase, a promotion of cystine uptake by formation of γ-glutamylcystine can also be excluded” [14]. This study indicates that γ-glutamylcystine may not play a significant role in GSH regulation in neurodegenerative diseases.

The EAAT system is responsible for about 90% of total cysteine uptake in neurons, whereas cystine does not serve as a source for neuronal GSH synthesis [9]. While five EAATs are known to exist, EAAC1 is primarily responsible for cysteine uptake in neurons [9]. Approximately 20% of EAAC1 is located at the plasma membrane during basal conditions, but during times of stress EAAC1 translocates to the plasma membrane [15]. EAAC1 expression can be modulated by a variety of factors including serum- and glucocorticoid-inducible kinase, phosphoinositide-dependent kinase, and GTRAP3-18 (as reviewed in [8]). The importance of EAAC1 to support induction of GSH synthesis and cellular viability has been investigated recently. Watabe *et al*. reported that knockdown of GTRAP3-18 (a negative regulator of EAAC1) in HEK293T cells increases intracellular GSH, and conversely increasing GTRAP3-18 protein expression using methyl-β-cyclodextrin leads to a decrease in intracellular GSH content [16]. Additionally, knockdown of EAAC1 or increased expression of GTRAP3-18 lead to increased DNA fragmentation when in HEK293T cells treated with H_2_O_2_ [16]. Demonstrating the importance of EAAC1 in neuronal cells, *in vitro* partial knockdown of EAAC1 in primary neurons resulted in ~20% decrease in cysteine uptake and a 30% increase in cell death when these altered neurons were treated with H_2_O_2_ [17]. Furthermore, *in vivo* embryonic knockout of EAAC1 showed age dependent neurodegeneration and significant loss of GSH content [18]. For a more in depth review on neuronal GSH synthesis and its regulation refer to [8].

In astrocytes the primary precursor imported into the cell for GSH production is cystine. The transport of cystine into astrocytes is through the Xc^−^ transporter, which is a sodium-independent transport system sensitive to inhibition by glutamate. The Xc^−^ transporter is intimately involved in GSH synthesis [19]. The imported cystine is rapidly reduced within the cell to form cysteine. The cysteine can then be used to generate GSH, and during times of oxidative stress GSH production is increased [19]. In order to provide the necessary cysteine for GSH synthesis during stress, Xc^−^ levels can be increased. In cell culture induction of Xc^−^ has been stimulated by a variety of factors including low levels of oxygen and H_2_O_2_-induced stress (via glucose oxidase reaction with glucose in the media) [20,21]. Electrophilic agents such as diethyl maleate and the vicinal dithiol binding agent/reducing agent arsenite have been shown to increase transcription of the *xCT* gene, mediated through the interaction between the ERE and the transcription factor Nrf2 [19]. Functionally, the importance of the Xc^−^ transporter can be highlighted by a study by Shih *et al.* which reports that over expression of the xCT protein increases GSH content and protects primary astrocytes against H_2_O_2_-induced cell death [22]. Moreover, mice with functionally incompetent Xc^−^ (*sut*/*sut* mice) show pronounced brain atrophy around 3–4 months [22]. Thus, the Xc^−^ transporter appears to play an important role in GSH synthesis in astrocytes. For more detail on the properties of the Xc^−^ transporter and its regulation see reviews [23,24].

In addition to imported cyst(e)ine, the transsulfuration pathway may also contribute to the supply of intracellular cysteine for GSH synthesis in astrocytes [25]. In this process endogenous sources of sulfur (e.g., homocysteine) can be converted to cysteine via a series of redox sensitive enzymatic reactions that contribute to the generation of GSH [26]. It should be noted that that approximately 50% of liver GSH is derived from transsulfuration, and that the brain cystathionine-γ-lyase (an enzyme key to the transsulfuration pathway) is reported to be more than 100-fold less active than the liver enzyme [26]. It has been reported that deficiencies in the transsulfuration pathway cause increased ROS levels (reviewed in [27]). During basal conditions in astrocytes, somewhere between 20% and 40% of the GSH is made through use of the cysteine generated through the transsulfuration. While only representing a minor fraction of the GSH production at steady state, the importance of the transsulfuration pathway is reflected during times of stress. Indeed, use of diethylmaleate (a GSH depleting agent) on primary astrocytes results in an increase in cystathionine-γ-lyase [28]. As mentioned earlier the Xc^−^ exchanger is a key contributor to cellular GSH generation and cellular survival by facilitating cystine uptake. Damage to the Xc^−^ exchanger *in vivo* would likely necessitate reliance on cysteine generation intracellularly. Indeed, the protein level of cystathionine-γ-lyase in C6 glioma cells nearly doubled 48 h post inactivation of the Xc^−^ exchanger through use of either L-α-aminoadipate or l-β-*N*-oxalylamino-l-alanine [28]. Thus, transsulfuration can be an important process for maintaining GSH levels in astrocytes, particularly in times of oxidative stress. In contrast this pathway is essentially absent in neurons. A more in depth description of the transsulfuration pathway in astrocytes is provided in the excellent review by McBean [25].

As described above, the majority of studies of GSH homeostasis in the brain have been carried out with astrocytes or neurons; however, microglia are also integral to brain tissue. In this regard, studies of microglia have reported that the Xc^−^ transporter is present in microglia, and that EAAT proteins are not basally expressed. However, it has been reported that EAAT proteins can be induced in microglia by lipopolysaccharide [29,30]. It is noteworthy that microglia contain higher levels of GSH than neurons and astrocytes [31], but studies are still ongoing to determine the relative contributions of the Xc^−^ and EAAT transporters to supply precursors for GSH synthesis. More information on GSH homeostasis in microglia may be found in recent reviews [30,32]. 

## 3. Glutathione Transport across the Blood Brain Barrier

GSH and glucose are key molecules in the functional survival of the brain, but their concentrations and mode of transport into the brain differ remarkably. GSH is the major antioxidant in the brain cells at a concentration ~2 mM [1]. GSH in blood plasma, however, is present at much lower concentrations. For example, the average level of GSH in the plasma of humans has been estimated to be ~2 μM [33,34]. Thus, GSH concentration in brain cells is about 400-times higher than in blood. In contrast to GSH, glucose concentrations in the brain cells are about 20% of the glucose concentrations in blood [35]. 

**Figure 2:**
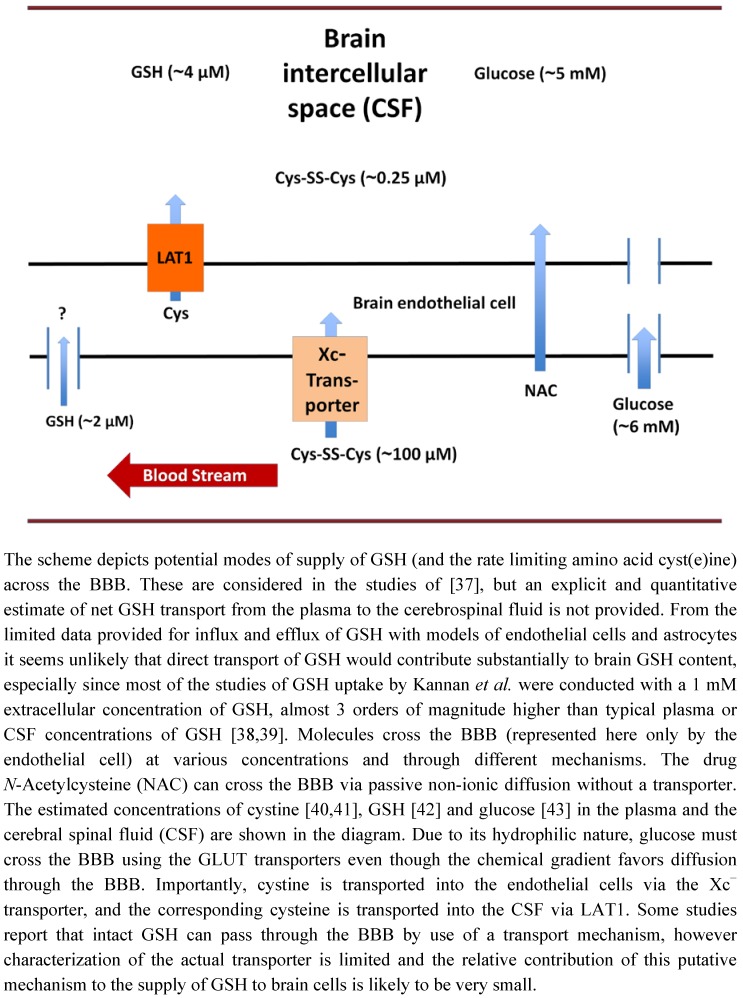
Transport of molecules across the blood brain barrier.

The blood brain barrier (BBB) is comprised of a layer of endothelial cells with tight junctions, surrounded by a sheath of astrocytes. The BBB functions as a selective barrier separating the brain from potential toxicants in the blood. The BBB has selective transport mechanisms to import necessary nutrients that cannot traverse the BBB by passive non-ionic diffusion, and P-glycoprotein transporters operate in the opposite direction to export non-essential metabolites and xenobiotics. Neither glucose nor GSH, both hydrophilic compounds, can passively diffuse through the lipid border of the BBB. Hence, penetration of glucose or GSH through an intact BBB implies specific transport mechanisms (Figure 2). Glucose is known to be transported into the brain by the GLUT family transporters [36]. In contrast, the concept of permeation of the intact GSH molecule through the BBB has been controversial and supportive data are limited. 

While some studies suggest that a specific GSH transport mechanism exists, the more common interpretation is that GSH transport across the BBB does not occur to an effective extent. Consistent with this negative conclusion, a study by Cornford *et al.* found negligible uptake of ^3^H-GSH into the brain after intracarotid injection [44]. Later a series of articles appeared that reported the existence of a GSH transport system. Thus, Kannan *et al.* reported that ^35^S-GSH, but not ^35^S-GSSG was able to pass into the brain within 15 s after intracoratid injection [38]. Importantly, the authors pretreated the rats with acivicin to inhibit GGT and prevent breakdown of GSH into its components (CysGly or Cys) that are transported across the BBB and readily enter the brain cells (Figure 1). In a subsequent study Kannan *et al.* reported that they were able to inhibit entry of ^35^S-GSH with structural analogs of GSH, but they reconfirmed that GSSG did not inhibit GSH entry [39]. The authors argued that the previous study by Cornford *et al.* did not take precautions to keep GSH in its reduced form, and that commercially available ^3^H-GSH was not pure [38]. Abbott *et al.* reported commercially available GSH at that time was only 63% pure [45]. While the report that a GSH transporter exists is provocative, the Kannan *et al*. studies overlooked some key points [38]. First, their criticism concerning oxidation of GSH in the Cornford study seems inaccurate. While some GSH may have been oxidized during the procedure, virtually all of the GSH would have to be oxidized to GSSG for no uptake to be observed, because GSSG is not inhibitory according to the reports of Kannan *et al.* themselves [38,39]. Impurities were likely in the ^3^H-GSH used for the initial study. However, these impurities would not alter entry through a selective transporter, unless the impurities were highly selective and highly potent inhibitors. Furthermore, Kannan *et al*. did not provide a quantitative analysis of how much GSH was actually taken up by the cells in these *in vivo* studies [38,39].

GSH transport has been investigated further by using two *in vitro* models of the BBB. Human cerebrovascular endothelial cells (HCEC) possess morphological and biological similarities to the BBB [46]. Using these cells, Kannan *et al.* reported uptake of ^35^S-GSH in both a Na^+^-dependent and Na^+^-independent manner [37], and estimated the maximum net rate of GSH uptake by the cells to be ~2.4 pmol/30 min/million cells. Considering that 10^6^ cells comprise an aqueous volume of ~0.01 mL, the reported uptake would correspond to a net change in intracellular GSH concentration of 0.24 μM. Efflux was reported to occur also, but at a slower rate that was inhibited by sodium. Since the total intracellular GSH concentration is in the millimolar range this change in concentration would seem to be inconsequential unless it served a microcompartment of the cells. As a mechanism of traversing the BBB, it also seems very limited because the net flux would be limited by the even slower efflux rate into the CSF. Nevertheless, these studies prompted a more recent study, which reported that construction of carrier molecules by conjugation with GSH via a peptidomimetic linker and a disulfide bond could enhance the uptake of adamantine and dopamine into Madin Darby canine kidney (MDCK) cells, another putative model of the BBB [47]. However, the latter results challenge the concept of a specific GSH transport mechanism because the adducted molecules constitute rather large alterations in the fundamental structure of GSH. While the concept of specific GSH transporters for direct access of GSH to the brain is intriguing, analysis of available data favor re-synthesis in the brain cells as the major source of GSH.

As discussed above, cysteine is the rate limiting amino acid in GSH synthesis; therefore the transport of cyst(e)ine into the brain is critical. Within the plasma, concentrations of cysteine (10–20 μM) are approximately 10 fold lower than the oxidized form cystine (100–200 μM) [40]. Using a mouse brain endothelial cell line Hosoya *et al. * determined that cystine is transported into brain endothelial cells through the Xc^−^ transporter [48]. As with astrocytes, the cystine is rapidly reduced intracellularly to cysteine to be used by the endothelial cells or pumped out into the intercellular space of the brain. Cysteine is transported out of the endothelial cells by the LAT1 subunit of System L [49,50]. Briefly, System L is part of the SCL7 family of Na^+^ independent heterodimeric amino acid transporters. It is composed of two subunits LAT1 and LAT2. Once in the CSF, cysteine can be taken up by neurons, pumped against the concentration gradient via the ASC system back into the endothelial cells or (oxidized to cystine) taken up by astrocytes and used for GSH synthesis. A thorough review of the unique properties of System L pertinent to the BBB has been presented recently [40]. 

## 4. Experimental Manipulation of Cellular GSH Content

Experimentally, intracellular levels of GSH can be altered by many approaches. Commonly used agents include: *N*-acetyl-cysteine (NAC), glutathione ether ester (GEE), diamide, buthionine sulfoxime (BSO), H_2_O_2_, and Xc^−^ transporter inhibitors. These compounds can be used to increase or decrease GSH content *in vitro* and *in vivo*. NAC can traverse the lipid membrane and be hydrolyzed to cysteine within the cell. The resultant cysteine can serve as a precursor for synthesis of more GSH. GEE is able to enter the cells, where the ethyl ester is hydrolyzed to yield more GSH [51]. Diamide is a selective oxidant that promotes conversion of GSH to GSSG and to protein mixed disulfides (protein-SSG), thereby depleting GSH content [52]. BSO inhibits the rate-limiting enzyme GCS, thereby blocking *de novo* synthesis of GSH in the cells. Natural utilization/degradation of the existing GSH then leads to its depletion over time. For example, ~80% loss of GSH is observed 24 h after BSO treatment in SH-SY5Y cultured nerve cells, and such depletion sensitizes the cells to oxidant-induced apoptosis [53]. Oxidizing agents like H_2_O_2_ are reported to induce increases in Xc^−^ transporter levels, resulting in increased intracellular cysteine and consequently increased GSH synthesis [23]. Xc^−^ transporter inhibitors (e.g., sulfasalazine and analogs) have the opposite effect, blocking import of cystine and resulting in limited GSH synthesis [54,55]. 

## 5. Glutathione Cycle

**Figure 3:**
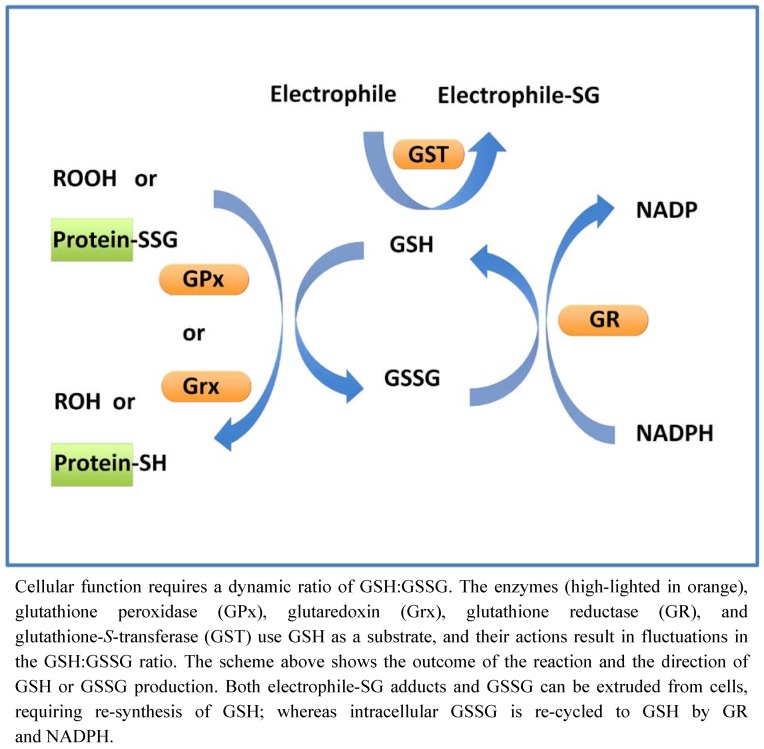
Maintenance of the glutathione cycle.

The importance of GSH derives primarily from two factors. First, it is the most abundant intracellular nucleophile and serves as substrate for many antioxidant and electrophile-scavenging enzymes. Secondly its unusual gamma amide linkage between glutamic acid and cysteine imbues unique specificity, and resistance to common peptidolytic degradation. Glutathione is present in the cell in reduced (GSH) and oxidized (GSSG) forms. The intracellular ratio of GSH:GSSG is typically >100:1, serving as an important redox buffer system for maintenance of the dynamic thiol-disulfide steady-state in cells. The reduced GSH serves as a substrate for enzymes that scavenge reactive oxygen species (ROS), inactivate electrophilic species, and restore reduced cysteine-thiol moieties on proteins, with the concomitant production of oxidized GSSG. All of these reactions serve to promote cell survival. The oxidized GSSG is recycled back to GSH, as depicted in Figure 3.

## 6. Glutathione-Dependent Enzymes

Since, GSH is a substrate for many important enzymes its cellular concentration must be maintained by re-synthesis as depicted above (Figure 1). Four distinct classes of GSH-dependent enzymes contribute to cellular redox homeostasis: glutathione peroxidases, glutathione transferases, glutaredoxins, and glutathione reductases. 

### 6.1. Glutathione Peroxidases

The removal of excess H_2_O_2_ protects cellular components from oxidative damage such as modifications of proteins and lipids, which can cause dysfunctions in cellular reactions and possibly cell death. Glutathione peroxidases (GPxs) reduce peroxide by catalyzing the conversion of reactive peroxides to their respective alcohols and water: [H_2_O_2_ (or ROOH) + 2GSH → 2H_2_O (or ROH + H_2_O) + GSSG]. GPx1 is ubiquitously expressed, and it is the most abundant glutathione peroxidase that functions to scavenge H_2_O_2_. As depicted in the equation above, the reduction of H_2_O_2_ results in the formation of one GSSG molecule per one molecule of H_2_O_2_ reduced. Likewise GSSG is produced when GPx4 (lipid-peroxide-selective GPx) acts on lipid peroxides. In addition to the GPxs, catalase and the peroxiredoxin enzymes also serve as catalysts of H_2_O_2_ scavenging (for a review on the relative contributions of the peroxide scavengers in neuronal peroxide metabolism, see [56]).

### 6.2. Glutathione *S*-Transferases

Glutathione *S*-Transferases (GSTs) are a family of enzymes, which catalyze the specific adduction of GSH to electrophilic substrates. The glutathionyl adducts are exported from cells by P-glycoprotein transporters, serving to eliminate potentially cytotoxic agents. This GSH conjugation is an important mechanism for xenobiotic transformation and cancer chemotherapeutic resistance (reviewed in [57]). As described below, GSTs have also been implicated as catalysts of GSH adduction to cysteine residues of specific proteins (protein-*S*-glutathionylation). 

### 6.3. Glutathione Reductases

A proper [GSH]:[GSSG] ratio is critical for cellular redox homeostasis. Glutathione disulfide reductase (GR) is critical to maintaining reduced GSH. GR catalyzes the NADPH-dependent reduction of GSSG to GSH (GSSG + NADPH + H^+^ → 2GSH + NADP^+^). Through coupling of the actions of GPx and GR, H_2_O_2_ is removed, GSH is maintained, and NADPH is consumed (Figure 3). At the cellular level, replenishment of the NADPH is dependent on the metabolism of glucose (e.g., though the action of glucose-6-phosphate dehydrogenase).

### 6.4. Glutaredoxins

Glutaredoxins (Grxs, also known as thioltransferases) specifically catalyze the displacement of GSH from protein-glutathione mixed disulfides (protein-SSG) through a thiol disulfide exchange reaction (protein-SSG + GSH → protein-SH + GSSG). As described under protein-*S*-glutathionylation (below), the reversible formation of protein-SSG can serve to modulate the functions of specific proteins that serve as intermediates in signaling pathways that mediate cell survival *versus* cell death. Analogous to phosphatase action in kinase signaling cascades, glutaredoxin can regulate the steady-state levels of protein-SSG signaling intermediates. Glutaredoxins display an exquisite specificity for GSH-containing protein mixed disulfides, and GSH is also the preferred substrate for reducing the Grx-SSG catalytic intermediate, resulting in net formation of GSSG (for review of Grx catalysis see [58]). Like glutathione peroxidase, coupling of the catalytic cycle of Grx to glutathione reductase and NADPH replenishes GSH (Figure 3).

## 7. Oxidative Stress and Dysregulation of Thiol Homeostasis

Oxidative stress has been implicated as a factor mediating initiation and progression of many neurodegenerative diseases, including Alzheimer’s disease (AD), Parkinson’s disease (PD), Huntington’s disease (HD), amyotrophic lateral sclerosis (ALS/Lou Gehrig’s disease), and Friedreich’s ataxia (FA) [59,60,61,62,63,64]. Oxidative stress is a condition of overabundance of reactive oxygen species and/or reactive nitrogen species (RNS). ROS are relatively unstable molecules (superoxide, hydrogen peroxide, hydroxyl radical) derived from molecular oxygen by electron addition, and RNS refers to nitric oxide (generated by nitric oxide synthetases) and its derivatives. Accumulation of excess ROS/RNS can oxidize cellular components; including lipids, proteins, and DNA, leading to impaired function and eventually cell death. Oxidative stress damage *in vivo* has been attributed to H_2_O_2_ or superoxide reacting with divalent metal ions to form hydroxyl radicals. Hydroxyl radicals, however, are such highly reactive species that they probably are not the proximate mediators of tissue damage. More likely, two electron-based reactive species like H_2_O_2_ (and peroxynitrite (see below)) react with protein-cysteine residues to mediate the damage that leads to cell death. Thus, emphasis in this review is focused on disruption of protein thiol homeostasis, which is mostly mediated by non-radical reactions, as a major basis of neurodegenerative disease. This point of view is reinforced by a compelling analysis of how covalent modifications of cysteine resides can more effectively account for tissue damage due to oxidative stress in human diseases than free radical-mediated reactions [65]. Thus, thiol homeo-stasis involving GSH is a major theme of this review. An alternative perspective based on studies with yeast was recently presented [66], where GSH was suggested to be more important for iron metabolism than thiol homeostasis. However, this provocative viewpoint warrants further investigation in broader contexts. The magnitude to which ROS play a role in neurodegenerative diseases may be due in part to the extensive production of ROS in the brain. Although small, the brain uses about 20% of the body’s oxygen supply [67], and a considerable amount is converted to ROS. Moreover, relative to other organs the brain has lower levels of ROS scavenging enzymes and a richer supply of unsaturated lipids that can propagate damage through reactive aldehyde products of lipid peroxidation [10]. ROS generation in the brain comes from multiple sources. Leakage of electrons to molecular oxygen at various points in the mitochondrial electron transport chain is the main source of ROS [68]. Complex I (NADH ubiquinone oxidoreductase) and complex III (ubiquinone-cytochrome C oxidase) produce superoxide and consequently peroxide as by-products of their enzymatic activities [69]. Additionally, H_2_O_2_ in the brain is a natural product of monoamine oxidase (MAO) reactions. The MAO enzymes catalyze the oxidation of neurotransmitters, thereby terminating their activity. For example, dopamine is oxidized by MAO-B with the concomitant conversion of O_2_ to H_2_O_2_ [70]. It is especially noteworthy that dopamine oxidation via MAO or autooxidation occurs primarily in the substantia nigra where oxidative stress is associated with loss of neurons, the pathological basis for Parkinson’s disease. Additional sources of ROS (specifically superoxide that dismutates to produce H_2_O_2_) include the NADPH oxidase enzymes (NOX) [71] and other flavoenzymes. ROS-mediated damage has been cited as a probable contributing cause for all of the common neurodegenerative diseases, implicating that the natural antioxidant defense mechanisms are overwhelmed in these cases.

**Figure 4:**
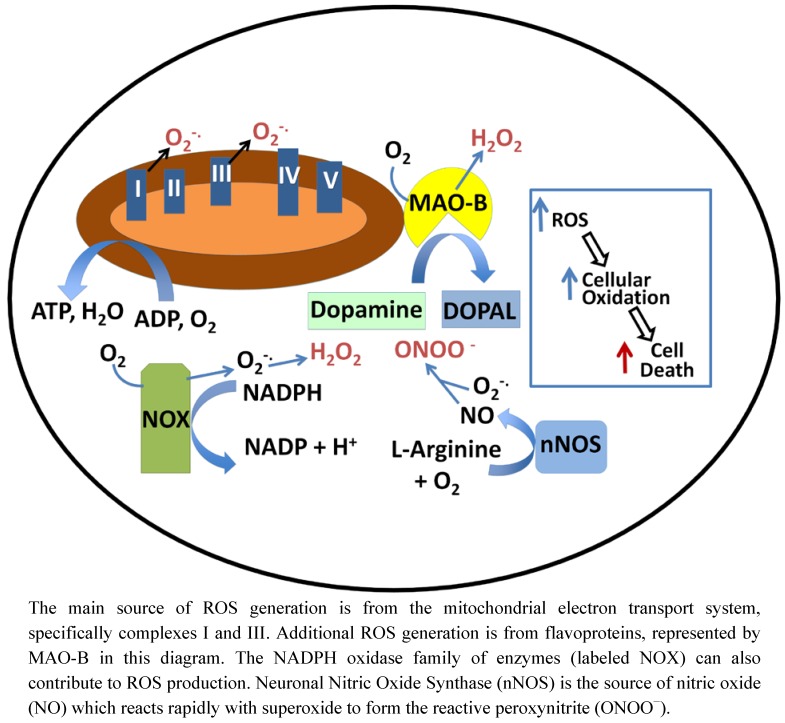
ROS production within cells.

Besides sources of ROS generation, the brain also has an abundant supply of nitric oxide (NO) due to the presence of neuronal NO synthase (nNOS). Unlike hydroxyl radical, superoxide and NO are not so reactive, but the radical recombination reaction between these two species produces the toxic oxidant peroxynitrite (ONOO^−^) at a rate one million times faster than OH-radical formation; and the more stable ONOO^−^ can diffuse 10,000-times farther than OH-radical [72]. Like ROS, ONOO^−^ leads to the oxidation of proteins, lipids, and DNA, but also mediates nitration of tyrosine residues. In turn, lipid peroxidation leads to formation of reactive aldehydes such as 4-hydroxynonenal (4-HNE) that covalently modifies cysteine residues via Michael addition. For example, 4-HNE inhibits the activity of GPx and thereby exacerbates oxidative stress. Figure 4 provides a schematic representation of various intracellular reactions that generate potentially cytotoxic ROS.

## 8. Mitochondrial Glutathione: Transport and Regulation of Apoptosis

GSH is synthesized in the cytoplasm of cells and must be transported into the mitochondria. The transport of GSH into the mitochondria is critical as GSH impacts apoptosis and cellular survival. The mitochondrial GSH accounts for approximately 15% of cellular glutathione. In general the mitochondrial GSH:GSSG ratio is greater than that of the cytosol, resulting in a more reducing environment [73,74]. One study of the GSH and GSSG levels in rat mitochondria reported that liver GSH concentration was 8.4 μM and GSSG was 0.02 μM, corresponding to approximately 250:1 GSH:GSSG ratio [75]. However, in brain mitochondria the GSH was reported to be 5.5 μM and GSSG 0.09 μM, giving an approximately 50:1 GSH:GSSG ratio [75]. The reason for this difference in redox ratio between tissues is not known, and should be investigated further. 

Two major GSH transporters have been identified: the 2-oxoglutarate carrier and the dicarboxylate carrier (reviewed in [76]). It is unclear currently as to which of these transport mechanisms is the predominant system for importing GSH into neuronal tissue. Kamga *et al*. reported western blot analyses showing that both the 2-oxoglutarate carrier and the dicarboxylate carrier are found in neuronal and glial cells of mice [77]. Additionally, they reported ~40% loss of the glutathione when cortical mitochondria were treated with a dicarboxylate carrier inhibitor; whereas only ~3% loss was observed with a 2-oxoglutarate carrier inhibitor [77]. These results demonstrate that the dicarboxylate carrier is the major transporter of GSH into the cortical mitochondria. However, mitochondria from granule neurons may present a different perspective. Wilkins *et al.* showed that BCL-2 co-immunoprecipitates with the 2-oxoglutarate carrier in primary cerebellar granule neurons [78]. Additionally, dual overexpression of the 2-oxoglutarate carrier and BCL-2 in CHO cells led to a substantial (~25%) increase in mitochondrial GSH [78]. Although the increase in mitochondrial GSH was found in CHO cells under overexpression conditions, the co-immunoprecipitation in primary cerebellar granule neurons suggests that the 2-oxoglutarate carrier in concert with BCL-2 may play a role in mitochondrial GSH uptake in neuronal cells; but further study is necessary.

Oxidative stress is known to induce apoptosis, and mitochondrial GSH status has become recognized as an important determinant in apoptosis. Work by Colell *et al.* reported that selective loss of hepatocyte mitochondrial GSH in ethanol-fed rats causes sensitization to TNF-α [79]. The hepatocytes from the ethanol-treated rats displayed approximately a 25% increase in cell death compared to control, and this increase in cell death could be ablated by incubation of the primary hepatocytes with GSH-ethyl ester prior to TNF-α addition [79]. Furthermore, the sensitization to TNF-α was recapitulated in control hepatocytes in which approximately 70% of mitochondrial GSH was depleted compared to approximately 25% of cytosolic GSH depletion using 3-hydroxyl-4-pentenoate [79]. Additional support for mitochondrial GSH in regulation of apoptosis is conveyed by the report (described above) that overexpression of the 2-oxoglutarate carrier and/or BCL-2 in CHO cells protects against H_2_O_2_-induced cell death [78]. While the studies described above implicated a critical anti-apoptotic role for mitochondrial GSH in non-neuronal tissue, Muyderman *et al.* reported that treatment of rat primary astrocytes with ethacrynic acid resulted in loss of virtually 100% of the mitochondrial GSH in 15 min [80]. Ethacrynic acid treated astrocytes were subjected to 3-morpholinosydnonimine (a peroxynitrite generator) and showed a 3 fold increase in LDH release (indicating cell death) and ~40% decrease in ATP production (indicating mitochondrial dysfunction), compared to controls [80]. Thus, mitochondrial GSH in astrocytes appears to be vital for defense against oxidant-induced cell death.

Apoptosis is key to neurodegenerative disease progression, and there is evidence that mitochondrial GSH may play a key role in regulating apoptosis. To further understand the role of mitochondrial GSH in neurodegenerative diseases, more direct studies involving cell lines and primary cells are needed. If alteration of mitochondrial GSH is established as a key factor in neurodegeneration, then studies seeking inducers of the 2-oxoglutarate carrier and/or the dicarboxylate carrier would be valuable. It is noteworthy that the genes for both the 2-oxoglutarate carrier and the dicarboxylate carrier have transcription factor Jun binding sites near the promoter (UCSC Genome Browser). Therefore targeting Jun may be an option for increasing expression of these carriers. 

## 9. Protein Degradation and Aggregation

In addition to ROS damage and apoptosis, another hallmark of neurodegenerative diseases are dysregulation of protein degradation and formation of protein aggregates. Protein aggregates have been linked to AD, PD, HD and other neurodegenerative diseases (for an excellent review on neurodegenerative disease and protein misfolding see [81]). Protein aggregation appears to be caused by an aberrant amount and/or modification of particular proteins in the cells. The perturbation due to changes in protein level/modifications is often caused by dysfunction of protein degradation complexes, leading to abnormal protein turnover, which has been implicated in many neurodegenerative diseases [82]. In this regard, the GSH-dependent antioxidant network has been implicated in regulation of protein degradation pathways involved in regulation of cell survival [83,84]. 

The protein degradation process removes misfolded, damaged, or otherwise non-functional proteins. Degradation can occur through either the ubiquitin/proteasome system or by autophagy. Proteins bound for the proteasome are primed with ubiquitin molecules by an ubiquitin ligase targeting system [85]. These proteins are then shuttled to the proteasome where they are broken down catalytically into small peptide fragments. Autophagy is a cellular system that uses the acidic lysosomes to break down proteins during times of starvation and when the ubiquitin proteasome is dysfunctional [86]. Autophagy can also be a mechanism of induced cell death. Dysfunctions in the ubiquitin/proteasome system and in autophagy have been implicated in neurodegenerative diseases [86].

Misfolded proteins are hazardous to the cell, and need to be either refolded properly or degraded. Folding of proteins occurs in the lumen of the endoplasmic reticulum (ER) [87], where chaperone molecules assist in configuring nascent polypeptide chains into stable three-dimensional structures. Formation of disulfide bonds is critical in folding and maintaining the proper 3-dimensional conformations of many proteins. Accordingly, protein disulfide isomerases catalyze disulfide bond formation and isomerization, facilitating proper pairing of cysteine residues [88]. Unlike most of the cell (which maintains a 100:1 GSH:GSSG ratio), the ER has an unusually oxidative environment estimated at a 5:1 GSH:GSSG ratio, and protein disulfide isomerase catalysis is enabled by this oxidative environment [89,90]. Before proteins leave the ER they must pass checkpoints for correct folding or be returned for re-folding. Proteins unable to be refolded properly are ubiquitinated and sent to the proteasome for degradation. In general the phenomenon of dysregulated protein folding in the ER is called ER stress that triggers the so-called unfolded protein response that can lead to cell survival or commitment to apoptosis. The role of ER stress-induced apoptosis has been reviewed recently [91].

When misfolded proteins are not adequately removed from cells, protein aggregation may occur. Particular types of protein aggregates have been identified as hallmarks of some neurodegenerative diseases: AD (amyloid beta plaques), PD (alpha synuclein-containing Lewy bodies), HD (huntingtin protein aggregates), and ALS (SOD1 aggregates) [92,93,94,95]. Irreversibly misfolded proteins are usually ubiquitinated to target them for degradation. The addition of the ubiquitin moiety is facilitated by E3 ligase enzymes that are highly specific for distinct protein families or individual proteins. Modification/deactivation of E3 ligases would disrupt degradation of their protein substrates. For example, TRAF6, a molecule containing E3 ligase properties, is regulated through *S*-glutathionylation [96], suggesting that alterations in glutathionylation status under oxidative stress would impair clearance of TRAF6-dependent substrates, and indeed TRAF6 has been implicated in Lewy body formation in PD [97]. 

**Figure 5:**
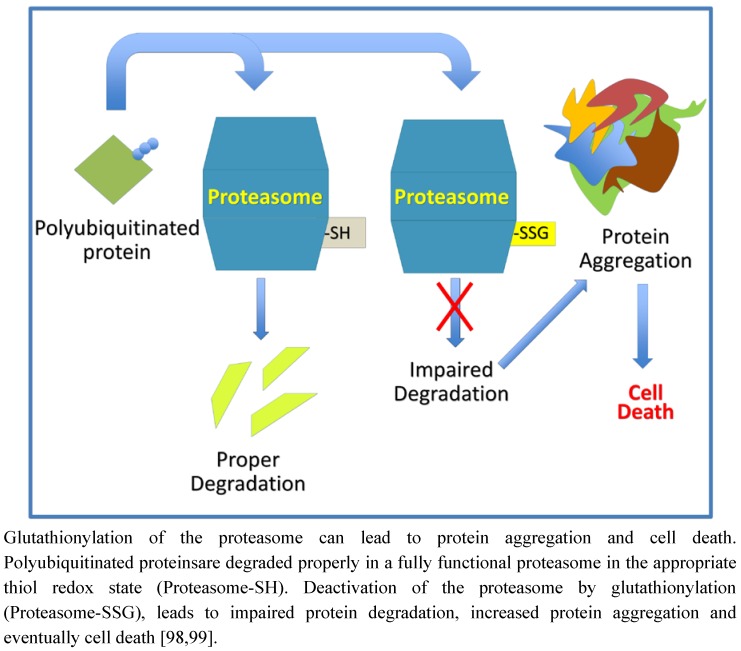
A role for protein *S*-glutathionylation in regulating protein aggregation.

Not only can the targeting mechanism (E3 ligases) of protein degradation be regulated by glutathionylation, but also so can the major protein complex responsible for protein degradation, the proteasome. Once ubiquitinated, proteins are transported to the proteasome where they are degraded. The catalytic activity of the proteasome has been reported to be inhibited by glutathionylation of the 20S subunit [98,99]. Inhibition of proteasome activity would lead to protein build up, formation of aggregates and potentially cell death. Figure 5 depicts a scenario by which disruption of normal protein degradation via *S*-glutathionylation could lead to protein aggregation and cell death. 

## 10. Protein *S*-Glutathionylation in Cellular Homeostasis and Regulation

As described in several contexts (above), protein *S*-glutathionylation is a specific posttranslational modification resulting from the disulfide adduction of GSH to a reactive cysteine on a target protein. This glutathionyl disulfide can serve to protect proteins from irreversible cysteine oxidation and/or serve as a functional modification analogous to phosphorylation [100]. 

The most likely mechanism for *S*-glutathionylation involves oxidation of a protein-cysteine moiety by H_2_O_2_, resulting in sulfenic acid formation (protein-SOH). This sulfenic acid is then converted to a mixed disulfide (protein:SSG) by a reaction with GSH causing the release of water. It is also been reported that the conversion of protein-SOH to protein-SSG could be catalyzed by a glutathione-*S*-transferase enzyme specifically delivering GSH to the protein-SOH moiety [101] (alternative mechanisms for protein *S*-glutathionylation are reviewed in depth in [102]). Regardless of mechanism, the conversion of the protein-sulfenic acid to protein-SSG serves as a protective mechanism which blocks further oxidation of the cysteine residue to sulfinic (protein-SO2H) or sulfonic (protein-SO3H) acids, which are irreversible modifications leading to protein degradation. Protein-SSG formation is specifically and efficiently reversed by glutaredoxin [58], restoring protein-SH when the oxidative stress subsides [103,104]. 

In addition to protein protection, *S*-glutathionylation is recognized as a mechanism of redox signal transduction and dynamic regulation of protein function. Analogous to phosphorylation of serine, threonine, and tyrosine residues, glutathionylation of cysteine residues can enhance or inhibit the activities of different proteins, promoting subcellular translocation or changing protein degradation patterns. Over the past decade, many proteins have been identified whose functions are changed by site-specific *S*-glutathionylation. However, demonstration of protein glutathionylation and functional alteration of proteins *in vitro* implies but does not document an intracellular regulatory mechanism. For glutathionylation of a particular protein to be considered physiologically relevant, it must occur intracellularly under natural conditions and elicit a physiological response (see previous reviews [103,105]). *S*-Glutathionylation plays an important role in cellular homeostasis and regulation of the balance between cell survival and cell death, and it appears to be one of the important manifestations of changes in GSH and GSSG content in the cells. The impact of alterations in protein *S*-glutathionylation in neurodegenerative diseases is discussed for specific examples in this document, and it is reviewed in more detail in [104]. 

## 11. Impairment of Glutathione Homeostasis in Neurodegenerative Diseases

GSH, the GSH-dependent enzymes, and redox regulation involving *S*-glutathionylation in signal transduction and sulfhydryl homeostasis are all considered to be key determinants of antioxidant protection against neurodegeneration. Dysregulation of the GSH-based antioxidant network in any way may contribute to the initiation and progression of the neurodegenerative diseases. In this section, examples of such dysregulation are discussed in the context of common neurodegenerative diseases: Parkinson’s disease, Alzheimer’s disease, Huntington’s disease, Friedrich’s ataxia, and amyotrophic lateral sclerosis (ALS/Lou Gehrig’s disease). Table 1 provides an outline overview of reports pertinent to this consideration. 

**Table 1:** Alterations in glutathione homeostasis that may contribute to neurodegenerative diseases.

Disease	Reported Changes in Patient Glutathione Homeostasis	Reported Alterations in Glutathione Related Enzymes	ROS Implicated in Disease	Reported Protein Aggregation Associated with Disease	Reported Protein Glutathionylation Associated with Disease
Parkinson’s Disease	Decrease in substantia nigra GSH [106]	GST-P1 (pi) mutation is associated with increased PD susceptibility [111]	Environmental factors that cause PD lead to oxidative damage [64]	α-Synuclein aggregates [92]	NADP-dependent isocitrate dehydrogenase [115]
Alzheimer’s Disease	Decrease in erythrocyte glutathione [107]	GPx1 mutation may be a risk factor for AD [112]	Beta amyloid may lead to mitochondrial instability, leading to increased ROS production [63]	Beta-amyloid, Tau aggregates [93]	Tau [116]
Huntington’s Disease	Decrease in plasma GSH [108]	Decreased GPx activity in erythrocytes [113]	Increased ROS production in mutant huntingtin-containing cells treated with thapsigargin [61]	Huntingtin aggregates [94]	NR
ALS	Decrease in erythrocyte GSH [109]	Decreased GST pi (P1) expression in motor brain cortex [114]	Mutated SOD1 increases ROS levels [62]	SOD1 aggregates [95]	SOD1 [117]
Friedreich’s Ataxia	Decrease in free glutathione in erythrocytes [110]	NR	FA cells show an increased sensitivity to oxidative damage [60]	NR	Actin [118]

NR = not reported.

### 11.1. Parkinson’s Disease

PD is the second most common neurological disorder, affecting over one million people in the USA alone. Characterized by unsteady gait, resting tremor and rigidity, PD is a physically debilitating disease [119]. PD appears to be caused by a selective loss of dopamine-producing neurons in the substantia nigra. The pars compacta subregion contains the greatest density of dopaminergic neurons responsible for propagating the signals that control voluntary movement. The neuronal death and progression of PD is thought to be induced largely by ROS [64]. ROS involvement in PD progression is consistent with the concept that impairment of the protective roles of GSH and associated enzymes could lead to PD initiation or exacerbation. For example, in post mortem brain tissue from PD patients, samples showed decreased content of GSH compared to controls [106]. The authors reported that postmortem times were the same for the PD patient’s and control samples [106]. In cellular models of PD, sensitivity to ROS has also been documented. For example, lentiviral overexpression of GPx1 in SH-SY5Y cells provided robust protection against 6 OH-DA challenge [120]. Using the same vector, mice overexpressing GPx1 also showed a significant protection against 6 OH-DA induced dopaminergic degeneration [120].

Mutations in a number of specific genes have been associated with familial PD. Remarkably; many of the corresponding proteins have been shown to have cysteine residues sensitive to oxidation. Thus, redox sensitive proteins associated with familial PD include α-synuclein, Parkin, and DJ-1. α-Synuclein was the first gene correlated to familial PD. Its aggregation is involved in Lewy body formation and subsequent neuronal cell death [121]. Oxidized glutathione (GSSG) is known to accelerate this aggregation [122], and neuronal death associated with α-synuclein in *Drosophila* can be rescued by interventions that increase reduced glutathione [123]. DJ-1 is an important cellular redox sensor, and mutations in DJ-1 resulting in loss of enzymatic function have been shown to cause autosomal recessive PD [124]. Loss of functional DJ-1 protein by cysteine modification could recapitulate the effects due to loss of function mutations. In this regard, knockdown of Grx1, the primary enzyme responsible for deglutathionylation, was found to result in decreased DJ-1 protein content without affecting mRNA levels [125]. These results suggest that impairment of DJ-1’s antiapoptotic activity by *S*-glutathionylation of oxidant-sensitive cysteines may be critical in DJ-1 induced PD; however, further studies are needed to verify this interpretation. Parkin is an E3 ligase, and similar to DJ-1, mutations have been shown to cause autosomal recessive PD. Parkin has cysteine residues that are susceptible to oxidative modification with concomitant inhibition of ligase activity [126]. *Drosophila* lacking Parkin display increased neuronal degeneration compared to controls. Moreover, induction of an antioxidant response that leads to increased GSH rescues the dopaminergic neuronal degeneration [123]. These studies indicate that oxidative modifications affect proteins identified with familial PD. Certainly, more studies are needed with various models of PD to elucidate the complex relationships between oxidative modifications of specific proteins and onset and progression of PD. Such studies are expected to shed light on PD pathogenesis and reveal connections between heritable and sporadic PD.

Besides the coincidence of oxidant sensitivity with proteins whose mutations are linked to PD, there are also reports of GSH-dependent enzymes whose mutations are correlated with PD. For example, GST-P1 is an isoform of GST involved in many cellular processes including xenobiotic clearance and apoptosis [127,128], and its mutation has been correlated with increased risk of PD [54]. Thus, Vilar *et al.* determined that 69-year old or older patients in their cohort who were heterozygous for the isoleucine-105 to valine substitution in GST-P1 had a doubled risk for PD [111]. Lymphocytes from patients with either Ile/Val or Val/Val genotypes at position 105 had significantly decreased GST activity compared to patients with the normal Ile/Ile genotype [129]. Additionally, lymphocytes isolated from patients harboring the Ile-105 to Val mutation had increased DNA damage [129,130]. These reports suggest that GST-P1 plays an important role in dopaminergic nerve cell survival. Considering the primary function of this enzyme, one might speculate that it plays a key role in scavenging electrophilic oxidative metabolites of dopamine (e.g., dopaquinone), serving to protect neuronal proteins from irreversible Michael adduction reactions. Future studies are necessary to test this hypothesis. 

*In vitro* and *in vivo* studies have started to delineate molecular mechanisms that link alterations in GSH-associated functions and cell death in PD. Engineered variation in GSH content itself has been associated with neuronal death. Using rat nigral dopaminergic cells, Garrido *et al.* showed that knock down of GSH synthesis by viral based RNAi directed against GCS, or over-activation of GSH synthesis, increased neuronal cell death [131]. These observations indicate that GSH content is finely regulated in healthy neurons, so that a shortage or an overabundance may jeopardize cell survival. Previous data reported by Sabens *et al*. showed that GSH depletion by BSO (a GCS inhibitor) substantially increased sensitivity of SH-SY5Y dopaminergic cells to L-DOPA-induced apoptosis [53]. These authors also reported that L-DOPA treatment led to inactivation of Grx1 in the cells, likely due to dopaquinone adduction of Cys-22 of the enzyme, as documented *in vitro* by mass spectrometry. A key role for Grx1 in neuronal cell survival was further supported by increased apoptosis in untreated cells in which Grx1 was knocked down by siRNA [53], suggesting that disruption in the regulation of protein glutathionylation may predispose these dopaminergic cells to apoptosis [132].

Consistent with the hypothesis of altered thiol-disulfide homeostasis (above), glutathionylation of isocitrate dehydrogenase is reported to occur in mouse brains after treatment with MPTP, linking it to a model of PD [115]. Furthermore, glutathionylation of isocitrate dehydrogenase inactivates the enzyme [115]; and knockdown of isocitrate dehydrogenase expression by siRNA increases susceptibility to apoptotic stimuli [133]. More recently, it has been reported that enzymatic inactivation of isocitrate dehydrogenase exacerbates (−)-epigallocatechin-3-gallate induced apoptosis in HeLa cells [134]. Thus, the enzymatic activity of isocitrate dehydrogenase appears to play an important role in regulating cell survival. Taken together these observations suggest that oxidant-induced deactivation of isocitrate dehydrogenase could contribute a pivotal role in PD. Inactivation of isocitrate dehydrogenase through glutathionylation is likely one of many examples by which oxidative stress induced protein modification of PD-linked proteins may alter function. Assessment of global changes in protein glutathionylation patterns in postmortem sample from PD patients compared to controls has not been reported. Such data could lead to new glutathionylated targets involved in PD pathogenesis. 

### 11.2. Alzheimer’s Disease

Alzheimer’s disease (AD) is currently the most common neurodegenerative disease. AD is characterized pathologically by beta-amyloid plaque formation and neurofibrillary tangles (tau containing moieties) in the brain. Symptoms of AD include memory loss and increasing dementia. Oxidative stress has been implicated in AD progression. This is supported by the observation that dissected post mortem AD brains show an increase in both nuclear and mitochondrial DNA oxidative damage in the cerebral cortex and cerebellum compared to age matched controls [135]. Additionally, amyloid-beta has been shown to be a pro-oxidant itself, and this feature of amyloid-beta has been proposed to be partially responsible for ROS generation [136]. This oxidation is thought to play an important role in neuronal death that leads to AD development and progression [137].

Analogous to patients with PD, mutations in GSH-dependent enzymes are reported to confer increased susceptibility to AD. For example, a polymorphism in the GPx1 gene (Pro198Leu) was identified as a possible risk factor in AD development [112]. *In vitro*, the GPx1 Pro198Leu mutant has been shown to have a small (10%) but significant diminution in enzymatic activity compared to WT GPx1 [138]. These data are consistent with a report that indicates that serum samples from AD patients have decreased GPx activity compared to healthy age-matched controls [139]. Additionally, polymorphisms in GST genes have been linked to early onset as well as faster cognitive decline in AD patients [140]. Blood samples from patients with severe AD were reported to have both a lower erythrocyte content of GSH and an increased level of GSSG compared to age-matched controls [107].

While the studies described above examined patient data, mechanistic studies with animal models and cell culture may also clarify the role of disrupted GSH homeostasis in AD. A recent study of mice harboring a mutation in the APP gene and a deletion in exon 9 of the presenilin-1 gene found that amyloid plaque deposits in the transgenic mice were increased at 6 months of age [141]. Notably, both genes are associated with familial AD. Consistent with increased oxidative stress and/or impairment of glutaredoxin activity, Zhang *et al.* reported an increase in protein-SSG levels in the cerebrum and cerebellum one month after birth in the same APP/presenilin-1 mutant mice [142]. The increase in protein-SSG in the cerebrum was still evident at the end of the 11-month monitoring period [142]. Although generalized increases in protein-SSG provide evidence for an oxidative stress condition, they do not provide much insight regarding mechanisms of progression of AD. Future studies would be more insightful by investigating the glutathionylation status and functional integrity of proteins directly implicated in AD initiation and progression. In this regard, human recombinant Tau protein, a building block in neurofibrillary tangles in AD, has been identified as being *S*-glutathionylated *in vitro* [116]. Considering the effects of glutathionylation that they observed on dimer equilibrium and rapid filament assembly of Tau–SSG, the authors speculated that a free thiol in Tau might retard filament assembly [116]. This speculation would imply that aberrant glutathionylation of Tau may result in an increased level of neurofibrillary tangles. However, further study is necessary to pursue this mechanism, including manipulation of the activity of glutaredoxin *in vivo*.

Increasing intracellular GSH as a protective approach against AD progression has shown promise. *N*-acetylcysteine can serve as a precursor for de novo synthesis of GSH. Accordingly, mice pretreated with NAC prior to intracerebroventricular injection of beta-amyloid showed an increased ability to learn and an improved memory function relative to controls [143]. As expected, GSH content was increased (almost double initial GSH levels) after *N*-acetylcysteine treatment. Additionally, protein and lipid oxidation were decreased [143]. The elevated GSH could have blocked the pro-oxidant effects of the beta-amyloid and prevented onset of the AD-like syndrome, or it might support more efficient repair of beta-amyloid mediated oxidative damage. Further work is needed to elucidate the mechanism. In another study, the model AD transgenic mice carrying mutations in APP and presinilin were given NAC orally for five months prior to the typical onset of plaque formation. The transgenic mice treated with NAC displayed lower amounts of lipid and protein oxidation and an increase in GPx and GR activities, reflecting a broad enhancement of antioxidant defenses. The mechanism for the induction of the GSH-dependent enzymes, however, is not known [144] and requires follow up studies.

Studies of cell culture models could provide mechanistic insights into the protective effects of NAC in the animal models. Thus, NAC administration to primary cortical neuron cultures decreased activity of typical signal transduction pathways that mediate cell death, including the MLK/JNK pathway [145]. Buildup of beta-amyloid molecules leads to plaque formation in AD, and the beta-amyloid is generated by γ-secretase-catalyzed cleavage of amyloid precursor protein (APP). Accordingly, limiting APP protein formation would be expected to decrease plaque formation. NAC administration was found to inhibit APP transcription to almost undetectable levels in SH-SY5Y cells; unfortunately, changes in the protein expression were not measured [146]. Down regulation of cell death pathways and decrease in APP synthesis may represent only a subset of the protective effects NAC administration confers in models of AD. 

The promising results of NAC in cell culture and mouse models of AD have led to human studies. Administration of 50 mg/kg/day of NAC daily for the duration of the study (6 months) resulted in favorable changes in some but not all cognitive measurements in human volunteers [147]. A case study by McCaddon and Davies reported that a 65-year-old man diagnosed with AD, severe dementia, and low GSH had an increase in word finding abilities and communication skills after NAC addition to his treatment regimen (duration of GSH regimen unknown) [148]. Although these studies represent a small subset of patients, they warrant consideration of a broader study to verify the effectiveness of NAC in AD. As of April 2012, a multi-site phase II clinical trial is recruiting patients to assess whether a nutraceutical (containing among other compounds 600 mg of NAC) maintains or improves cognitive performance in patients with AD or mild cognitive impairment (clinical trial identifier NCT01320527). Additionally, a study is in progress (clinical trial identifier NCT01370954) to test the effects of CerefolinNAC (a nutraceutical preparation containing L-methylfolate, B12 and 600 mg NAC) on “managing proper neuronal function in the brain” in patients with mild cognitive impairment, vascular dementia, or AD. With this combination (discussed in a recent review [149]), it appears that the l-methylfolate and NAC are added for different primary purposes. The rationale for NAC is straightforward, *i.e.*, to provide a precursor for GSH synthesis. The rationale for L-methylfolate appears to be as a precursor for vitamin B_12_ synthesis based on the sensitivity of B_12_ to oxidative stress. There is also the consideration that *S*-adenosyl methionine formation stimulated by L-methylfolate could serve as a source of homocysteine for the transsulfuration pathway to cysteine, but this is rather convoluted and certainly less direct than NAC. While the current studies are not ideal; *i.e.*, each supplement contains active compounds other than NAC, they may add support to previous indications of potential beneficial effects of NAC for AD patients. If benefits of these combination supplements are observed, then the contributions of each active ingredient toward AD relief need to be examined separately in appropriate models.

### 11.3. Huntington’s Disease

Huntington’s disease (HD) is a progressive neurodegenerative disorder characterized by rapid involuntary movements and dementia eventually leading to death. Its genetic cause is an autosomal dominant trinucleotide expansion (CAGCAG…) in the huntingtin gene. The underlying mechanism through which the expressed mutant huntingtin protein causes neuronal degeneration is still unclear, but oxidative damage has been implicated by post mortem studies reporting an increase in oxidized cellular components [150]. 

Similar to the other neurological diseases, GSH and GSH-dependent enzymes have been shown to be dysregulated. HD patients have decreased GSH content in plasma samples compared to age matched controls [108]. Additionally, GPx activity in erythrocyte samples from HD patients is reported to be lower than in age matched controls [113]. Another study, however, reported no difference in GPx activity in fibroblasts cultured from HD *versus* non-HD patients [151]. The reason for this discrepancy is unclear, but the underlying cause may reflect cell specificity, prompting measures of GPx activity in samples from post mortem brain to determine which of the surrogate samples may be more representative of the brain cells. Ideally, cell culture lines could be generated from neurons taken from HD and non-HD individuals.

Animal models of HD have given a small glimpse into what may occur in HD patients. Oddly, the HD mouse model R6/2 (a mouse model carrying the human HD gene plus roughly 120 CAG repeats [152]) showed a significant increase in GSH content in the mitochondria isolated from the cortex and striatum [153]. The authors hypothesize that the increased GSH could be a compensatory mechanism for increased ROS production, although the authors did not specifically measure ROS or other products of oxidative stress. Much like MPTP induction of PD symptoms, chemical induction of HD-like symptoms; *i.e.*, dyskinetic movements, can be achieved with the addition of 3-nitropropionic acid. Rats treated with nitropropionic acid showed a decrease in total (cytosolic plus mitochondrial) GSH and a decrease in GST function in the striatum, hippocampus, and cortex [154]. Specific levels of GSH in the mitochondria of the brain sections were not measured; hence it is unknown if these rats had changes in mitochondrial GSH consistent with what was previously reported [153]. However *in vitro* use of huntingtin knock-in striatal cell lines indicates that the depletion of GSH can be blocked by the administration of cystamine prior to 3-NP. This stabilization of GSH content resulted in less cell death. Importantly, BSO treatment (GSH depletion) in conjunction with cystamine resulted in no increase in cell survival strongly suggesting that normal GSH levels are the mediating survival factor [155]. However, the mechanism(s) by which maintenance or supplementation of GSH could protect against HD remain to be elucidated. 

In summary, studies of alterations in content and/or function of GSH and GSH-dependent enzymes in HD have reported dysregulation in these systems. While human subjects are not conducive to studying disease mechanisms, HD mouse lines are available that have not been exploited thoroughly in this regard. The conflicting results mentioned above regarding whether GPx activity is decreased in HD or not could be resolved by measuring the GPx activity in the nerves from recently deceased HD patients. Furthermore, generation of an HD mouse that lacks or overexpresses GPx would help discern if changes in GPx is important in HD. Generation of new models using the previously described HD mouse model along with mutations in GSH-dependent enzymes could serve as the basis for discovery of novel HD therapeutics.

### 11.4. Amyotrophic Lateral Sclerosis

Amyotrophic lateral sclerosis (ALS, Lou Gehrig’s disease) is a debilitating neurodegenerative disease that causes muscle atrophy and paralysis eventually leading to death. The cause of sporadic ALS is unknown, but since its discovery in 1993 many distinct mutations in the Cu, Zn superoxide dismutase (SOD1) gene have been repeatedly associated with familial ALS [156]. Different SOD mutations have been shown to result in distinct pathology. This phenomenon has been reported in mice harboring various SOD1 mutations, including Gly37Arg, Gly85Arg, and Gly93Ala. All three distinct mutations result in neurodegeneration [157,158,159]; however, it is intriguing that none of the mutations results in loss of enzyme function. Nevertheless, there are some differences among the mice bearing different SOD1 mutations. For example, the Gly93Ala mice but not Gly37Arg mice have increased levels of oxidized proteins in the spinal cord associated with disease progression [160,161]. Additionally, Gly37Ala mutant mice show presymptomatic mitochondrial defects while the Gly85Arg mutants do not [157,158]. Clearly, different mutations in SOD1 result in distinct pathogenesis. This is an important topic of discussion, and a more in depth review focusing on the biology of ALS including differences in SOD1 models is available [162].

Besides oxidative stress associated with diminished scavenging of superoxide, other studies have reported that depletion of GSH *in vitro* is accompanied by motor neuron cell death [163,164], simulating ALS. GSH and GSH-dependent enzymes appear to be dysregulated in ALS. For example, in one study erythrocyte GSH content in ALS patients was significantly lower than for age-matched controls; similarly, GR activity was also decreased [109]. The authors also reported that loss of GSH and GR activity progressed with time [109]. Studies of GPx activities in erythrocyte samples from ALS patients have given mixed results with some showing significant diminution while others showed no change [165,166], analogous to the situation described for HD (above). Another study reported that mRNA levels for GST pi were significantly down regulated in the spinal cord, motor cortex, and the sensory cortex of ALS patients [114]. Furthermore a cohort of patients with a unique haplotype signature in the glutathione synthase gene was found to correlate with ALS patients who had exposures to metals and chemical solvents [167]. The authors speculated that because the haplotype covers the entire gene, “changes anywhere [in the gene] may have a deleterious effect” [167]. These “deleterious effects” could manifest as mutations leading to decreased synthesis of GSH; however, GSH levels were not determined in this study. Hence it remains to be determined whether the cohort of patients with the unique GS-haplotype may have lower levels of total glutathione compared to other haplotypes. Thus, dysfunction of the GSH-dependent enzymes GR, GST, and GPx, or in the enzymes responsible for GSH synthesis may weaken antioxidant defenses further amidst the already compromised ROS-scavenging system, leading to greater damage and ultimately cell death.

Mechanistic studies over the past decade also speak to the role of GSH in progression of ALS. An intriguing study by Tartari *et al.* using NSC-34 cells containing an inducible Gly93Ala SOD1 vector, reported that short-term expression of Gly93Ala SOD1 resulted in a 30% increase in cellular GSH, while prolonged expression (14 passages) resulted in a 30% decrease in GSH [168]. Time dependent loss of GSH may explain part of the reason for latency of ALS onset. As discussed above, mitochondrial GSH likely plays an important role in neurodegenerative diseases. To investigate the role of mitochondrial GSH, stably transfected NSC-34 cells expressing mutant SOD1 Gly93Ala were reported to have depleted mitochondrial GSH [169]. This depletion of mitochondrial GSH likely contributed to increased apoptosis compared to controls when ethacrynic acid was added [169]. Additionally, Gly93Ala mice showed a decreased level of GSH and increased level of GSSG (indicating an oxidative environment), and increased active casapse 2 in motor neurons [164]. A more recent study indicated that depletion of GSH in an SOD1 Gly93Ala mouse model significantly shortened life span. The authors noted, however, that mice carrying a different mutation, SOD His46Arg/His48Gln, were not affected by GSH depletion [170]. Puzzled by these findings, the authors hypothesized that the difference might be related either to a differences in activity of the SOD mutants or to different subcellular localizations of the different SOD mutants. The differences in activity of the two mutant SODs seem to add to the puzzle rather than resolving it. Thus, SOD His46Arg/His48Gln is enzymatically inactive, but SOD1 Gly93Ala exhibits enzymatic activity. Thus, the antioxidant superoxide-scavenging activity of SOD1 paradoxically appears to be necessary for the pro-oxidant effect of GSH depletion, inducing cell death. This counterintuitive finding needs to be further investigated. As for the differential subcellular localization of SOD1, it is predominately localized in the cytoplasm, but can be found in the nucleus, ER, and mitochondria. SOD1-His46Arg/His48Gln, however, is thought to localize in the outer mitochondrial membrane; whereas SOD1-Gly93Ala is located in the intermembrane space. Clearly, not all mutations of SOD confer the same ALS phenotype, and differential localization may be an important determinant that requires further study.

Protein aggregation has also been suggested to play a key role in ALS [95]. SOD1 typically exists as a dimer; and this dimer formation is thought to protect the enzyme from damage and misfolding. However, SOD1 can self-associate into higher-level oligomers, ultimately forming cytotoxic aggregates [95]. A study in 2009 correlated occurrence of sporadic ALS with oxidative modification of SOD1, reporting that roughly 50% of ALS patients displayed S-glutathionylation on the Cys-111 residue of the enzyme [117]. Recently, this site-specific glutathionylation was shown to destabilize and alter the structure of the SOD1 dimer, increasing its dissociation constant over 1000-fold [171]. The resultant increase in monomeric SOD1 increases the likelihood of protein damage and aggregate formation. These studies suggest that glutathionylation of SOD1 (and other proteins) may contribute directly to progression of ALS.

Besides specific glutathionylation of SOD1, more widespread changes in protein glutathionylation may alter the functions of other proteins and contribute to exacerbation of the ALS condition, suggesting impairment of Grx function as well. Clearly these speculations require further study. 

### 11.5. Friedreich’s Ataxia

Friedreich’s ataxia (FA) is an autosomal recessive neurodegenerative disorder typically caused by trinucleotide expansion of GAA in the gene encoding frataxin. Although the mutation is analogous to that in HD, cellular consequences differ substantially. Thus, expansion of the frataxin gene causes iron accumulation within the mitochondria, likely creating a milieu that enhances oxidative stress [172]. This report and others led researchers to examine the status of GSH and GSH-dependent enzymes in FA.

Erythrocytes from FA patients were found to have a decreased concentration of reduced GSH but comparable levels of total GSH, compared to non-FA patients [110], consistent with an increase in protein-bound GSH (protein-SSG). A later study then documented that FA patients have increased spinal cord protein-SSG levels compared to healthy controls [173]. This increase in glutathionylated proteins reflects an increased oxidative environment and likely explains the diminished amount of free GSH in the FA patients. Analysis of a yeast FA model lacking the frataxin homologue showed a significant increase in GPx activity, suggesting an upregulation of antioxidant capacity; but the content of NADPH was decreased [174]. The diminished NADPH content indicates that its supply could not keep up with the demand for reducing equivalents. Consequently, the ability of GR to recycle GSSG to GSH would be limited by the supply of NADPH. Oddly, a separate study reported that overexpression of frataxin in 3T3 cells resulted in decreased susceptibility to *tert*-butyl peroxide, attributed to an increase in GPx activity [175]. Thus, in different contexts knockout and overexpression of frataxin gave comparable results. Whether these opposing results are due to cell specificity or organismal adaptation is unknown, but further study is necessary to elucidate these apparently contradictory findings.

Fibroblasts from FA patients were found to have impaired microfilament structures, and the authors concluded that aberrant S-glutathionylation of actin was responsible for the abnormal polymerization of actin [118]. This interpretation is supported by the previous observation that glutathionylation of actin at Cys-374 inhibits its polymerization [176]. Abnormalities in actin assembly have been linked to cellular death in other studies, including poly-glutamine induced neuronal dysfunction [177]. By analogy it seems that the actin dysfunction documented for FA patients may be a key contributory factor to neuronal cell death [177]. However, glutathionylation of other proteins may also contribute. Thus, consistent with the decreased content of reduced GSH in FA patient samples (cited above), a global increase in protein glutathionylation has been reported in FA samples [173]. 

It appears that the key to understanding and intervening therapeutically in FA may be accomplished through investigating in more depth the relationships among increased free iron, decreased GSH, and changes in glutathionylation and function of specific proteins, deciphering the benefit of reversal of these aberrations.

## 12. Glutathione in Food/Supplements

GSH plays a key role in protecting the body against ROS mediated damage. Increasing the levels of GSH within the brain may lessen the impact of oxidative stress associated with neurodegenerative diseases. Foods such as spinach, asparagus, and avocado contain mg quantities of GSH [178]. Unfortunately, consuming large quantities of these foods likely does not change the levels of GSH in the body. Witschi *et al*. showed that patients given 3000 mg of oral GSH (an amount exceeding 100-times a typical serving of avocado) led to no significant change in blood GSH or cysteine [179]. While consumption of pure GSH does not increase GSH levels; studies have reported that silymarin (milk thistle) injected into Wistar rats can increase GSH in the liver (brain tissue was not examined), curcumin (found in the spice turmeric) fed to Wister rats can increase GSH in the brain; and alpha lipoic acid (found in various vegetables), when added to SH-SY5Y cells increased intracellular GSH [180,181,182]. The mechanisms for the increases in GSH are unclear since they might be due to a variety of factors, including increased transcriptional activity of proteins used to synthesize GSH, increased translational activity, decreased degradation of GSH, increased reduction of GSSG, increased transport of precursors, *etc.* Curcumin has been reported to induce an increase in both mRNA and protein levels of glutamate-cysteine ligase (a key enzyme in the synthesis of GSH) [183]. The mRNA and protein increases were attributed to changes in transcription factor binding capacities in immortalized bronchial epithelial cells [183]. Whether analogous mechanisms are responsible for the observed increases in GSH levels in brain tissue remains to be determined. Nevertheless, all three compounds (silymarin, curcumin, and alpha lipoic acid) have been reported to display some protection *in vivo* in various neurodegenerative disease models, including PD (paraquot induced), AD (APPSw mice), ALS (Gly93Ala mice), and HD (R6/2 mice) [184,185,186,187]. Increasing GSH content represents one therapeutic strategy for treating neurodegenerative diseases, and while oral intake of GSH does not increase GSH levels in the body, it has become apparent that small molecules can induce changes in GSH content in animal models. The studies cited above provide limited examples of natural agents taken orally with potential protective effects for neurodegenerative diseases, possibly through modulation of GSH levels in the brain. Such studies on natural inducers of GSH are continuing and much remains to be learned about their efficacy and mechanisms of action. It is important to note however, that these studies were performed in animal models, and results cannot be extrapolated to human health until controlled trials on human subjects have been completed. The next section considers studies of direct intravenous administration of GSH to humans.

## 13. Glutathione as a Therapeutic Agent

Studies focusing on GSH and the GSH-dependent enzymes have revealed potential mechanisms and consequences of dysregulation of the GSH antioxidant network in neurodegenerative diseases. This realization has led some investigators to argue that GSH supplementation may be a viable treatment strategy for PD. As discussed above, oral GSH does not increase levels of GSH in the body; however, intravenous dosing of GSH apparently does [188]. In 1996 Sechi *et al.* undertook a non-blinded approach to dosing PD patients with intravenous GSH [189]. PD patients were given 600 mg GSH twice daily for 30 days. The authors reported an impressive 42% improvement in the Modified Columbia University Rating Scale (a clinical diagnostic test indicating the severity of PD). Such an improvement, however, was not observed in a randomized double-blind pilot study conducted by Hauser *et al*. in 2009 [190]. In the latter study patients were given 1400 mg of GSH or placebo intravenously three times a week for four weeks [190]. 

The article by Hauser *et al.* prompted contrasting letters to the editors published in the journal Movement Disorders. One letter by Okun *et al.* questioned the study’s statement concerning the possible symptomatic effect and the validity of combining the subscales (UPDRS and motor scores), and it raised questions about GSH crossing the BBB [191]. Additionally, Naito *et al.* questioned the safety of large doses of GSH [192]. In support of the study Dr. Sechi stated that research had shown that GSH does pass through the BBB [193]. Dr. Sechi referenced the study of engineered glutathione adducts as drug carriers by More *et al.* [47] that was described above. However, the Moore *et al.* data seem to conflict with the idea of specific GSH transport, because adduction of rather bulky molecules to GSH did not deter the GSH-conjugates from entering the cells. Therefore, while a specific transport mechanism has been proposed for GSH through the BBB, skepticism regarding the quantitative impact of that transport mechanism is warranted, as discussed above. Nevertheless, GSH itself would not have to cross the BBB to promote an increase in intracellular GSH content. As described (see Figure 1 and Figure 2 and accompanying text), cysteine levels limit GSH synthesis, and GGT enzymes break GSH into the CysGly dipeptide which has ready access into the brain cells where it serves as a precursor to cysteine and GSH [194]. Thus, precursors of GSH in the blood could lead to an increase in total GSH in the brain. Hence additional studies are warranted to distinguish the relative efficiency of GSH and its precursors to supplement the GSH content of neurons without eliciting toxic effects. 

*N*-acetylcysteine as a GSH precursor—while two clinical studies using intravenous GSH injection have shown opposing results, studies with NAC as a cysteine/GSH precursor have shown promising results in mice in both PD and AD models [144,195]. As discussed above, these results have led to ongoing clinical trials investigating the effects of NAC, which is already a FDA approved drug, in combination with other nutraceuticals for treatment of AD. Additionally, as of April 2012 two studies are actively recruiting PD patients for clinical trials with NAC (Clinical tracker numbers NCT01470027 and NCT01427517). Previously, an animal study by Andreason *et al.* reported an increased survival time (Kaplan-Meier curve indicated *p* = 0.01 treated *vs.* control) and increase in motor movement (via rotarod) in Gly93Ala mice (ALS model) treated with 1% NAC water starting from 4 to 5 weeks of age until death [196]. More recently, Sandhir *et al.* reported that NAC can reverse mitochondrial dysfunction in 3-NP treated rats [197]. These studies suggest a potential therapeutic benefit in ALS and HD with use of NAC; however, no clinical trials have been conducted using NAC in HD. One study has been completed in ALS. A clinical study addressing the therapeutic benefit of *N*-aceytlcysteine given at 50 mg/kg injected subcutaneously daily for 12 months showed no increase in survival in ALS patients compared to control [198]. Why the treatment in ALS was unsuccessful is unclear. The ongoing clinical trials with NAC for PD and AD, however, should help to assess the potential therapeutic effects of boosting intracellular GSH content without having to resolve the issue of direct transport of GSH into brain cells in certain neurodegenerative diseases.

## 14. Glutathione as a Biomarker?

Biomarkers for neurodegenerative diseases have been difficult to develop, but they represent an important area of research that could impact millions of patients quickly. Clearly, biopsy of nerves or the brain for tissue specific markers in a routine manner is not feasible. All of the neurodegenerative diseases discussed above had the common characteristic that changes in GSH content were observed in samples from affected patients compared to controls, regardless of whether they were post mortem brain samples or blood samples from live human subjects. However, the difficulty with GSH as a specific biomarker is that differences in GSH content could be due to a variety of dietary and environmental factors, and changes in GSH levels have been implicated also in other diseases ranging from diabetes to cancer. Thus, at best changes in GSH levels could only serve as confirmation of other more selective markers yet to be developed.

## 15. Conclusions

Over the past several decades the role of intracellular GSH status in neurodegenerative diseases has been studied intensively. Such research continues to provide mechanistic insights pertaining to the cellular dysfunctions of the neurodegenerative diseases, including Parkinson’s disease, Alzheimer’s disease, Huntington’s disease, amyotrophic lateral sclerosis, and Friedreich’s ataxia. Disruption in GSH homeostasis and modification of the enzymes that are dependent on GSH as a substrate have been linked to initiation and progression of the neurodegenerative diseases. The dysregulation of GSH and GSH-dependent enzymes induces a variety of cellular problems that can lead to mitochondrial dysfunction, accumulation of ROS/RNS damage, disruption of signaling pathways, protein aggregation, and ultimately cell death. It is certain that more research is needed not only to define more accurately how disruption of the network of GSH-dependent reactions leads to nerve cell damage, but also to discover new ways to prevent and/or reverse that damage and thereby develop more effective therapies for the neurodegenerative diseases. 
